# Risk of malignant lymphomas in patients with inflammatory bowel disease: a population-based cohort study

**DOI:** 10.1136/bmjgast-2022-001037

**Published:** 2023-05-04

**Authors:** Jingru Yu, Erle Refsum, Paulina Wieszczy, Lise M Helsingen, Vera Perrin, Amanda Högdén, Magnus Løberg, Johannes Blom, Michael Bretthauer, Hans-Olov Adami, Weimin Ye, Mette Kalager

**Affiliations:** 1 Department of Medical Epidemiology and Biostatistics, Karolinska Institutet, Stockholm, Sweden; 2 Clinical Effectiveness Research Group, Department of Transplantation Medicine, Oslo University Hospital, Oslo, Norway; 3 Clinical Effectiveness Research Group, Institute of Health and Society, Department of Health Management and Health Economics, University of Oslo, Oslo, Norway; 4 Department of Gastroenterology, Hepatology and Clinical Oncology, Centre of Postgraduate Medical Education, Warsaw, Poland; 5 Department of Clinical Science and Education, Södersjukhuset, Karolinska Institutet, Stockholm, Sweden; 6 Department of Epidemiology and Health Statistics & Key Laboratory of Ministry of Education for Gastrointestinal Cancer, Fujian Medical University, Fuzhou, Fujian, P.R. China

**Keywords:** lymphoma, inflammatory bowel disease, epidemiology

## Abstract

**Objective:**

To estimate the risk of non-Hodgkin’s lymphoma (NHL) and Hodgkin’s lymphoma (HL) in patients with inflammatory bowel disease (IBD).

**Design:**

We undertook a two-country population cohort study with all patients diagnosed with IBD in Norway and Sweden from 1987 and 1993 through 2015 and 2016, respectively, and analysed the risk of NHL and HL. In Sweden, we also analysed prescriptions of thiopurines and anti-tumour necrosis factor (TNF)-α therapy from 2005. We calculated standardised incidence ratios (SIRs) with 95% CIs using the general populations as reference.

**Results:**

Among 131 492 patients with IBD with a medium follow-up of 9.6 years, we identified 369 cases of NHL and 44 cases of HL. The SIR of NHL was 1.3 (95% CI 1.1 to 1.5) in ulcerative colitis and 1.4 (95% CI 1.2 to 1.7) in Crohn’s disease. We found no compelling heterogeneity in analyses stratified by patient characteristics. We found a similar pattern and magnitude of excess risks for HL. At 10 years, cumulative incidence was 0.26% (95% CI 0.23% to 0.30%) and 0.06% (95% CI 0.04% to 0.08%) for NHL and HL, respectively. Higher excess risks were found among patients with NHL with concomitant primary sclerosing cholangitis (SIR 3.4; 95% CI 2.1 to 5.2) and in those prescribed thiopurines alone (SIR 2.8; 95% CI 1.4 to 5.7) or with anti-TNF-α agents (SIR 5.7; 95% CI 2.7 to 11.9).

**Conclusion:**

Patients with IBD have a statistically significant increased risk of malignant lymphomas compared with the general population, but the absolute risk remains low.

**WHAT IS ALREADY KNOWN ABOUT THIS TOPIC**

- Several autoimmune diseases are associated with increased risk lymphoma, but the risk in patients with inflammatory bowel disease remains unclear due to inconsistent evidence.

**WHAT THIS STUDY ADDS**

- We present a large, population-based study with long-term and near complete follow-up.
- We demonstrate that the absolute risk of lymphoma in inflammatory bowel disease is low.

**HOW THIS STUDY MIGHT AFFECT RESEARCH, PRACTICE, OR POLICY**

- There is a need for even larger studies with detailed data on disease characteristics and treatment to quantify the risk associated with immunosuppressive therapies.
- Such studies need to consider the effect of confounding by indication, which requires valid data on disease activity.

## Introduction

An increased risk of Hodgkin’s lymphoma (HL) and non-Hodgkin’s lymphoma (NHL) has been documented in several autoimmune diseases, such as Sjögren’s syndrome, coeliac disease and rheumatoid arthritis.^1–4^ A link between disease severity and lymphoma risk has been established, and although the exact molecular mechanisms remain to be explained, longstanding chronic inflammation with B cell and T cell activation and/or stimulation are likely predisposing factors. Several mechanisms may be ongoing in parallel and the type of immune dysfunction involved in lymphoma development is thought to be disease specific, as different types of lymphoma are associated with the different disease entities.^4^ Immunosuppression is generally thought to play less of a role in lymphoma development in most autoimmune diseases, although such drugs may increase the risk of cancer through reduced immunological surveillance.^4^

Inflammatory bowel disease (IBD) is also an autoimmune disease characterised by chronic inflammation, but, contrasting to other autoimmune diseases, the available evidence on risk of lymphoma is scarce and too inconsistent to allow reliable quantification and guide patient care.^5 6^ Meta-analyses have concluded with both increased^7 8^ and no increased^9^ risk of lymphoma in patients with IBD treated with immunosuppressants. Limited statistical power, short follow-up, conflation of different haematopoietic malignancies, and lack of detailed data on immunosuppressive therapy are limitations of published studies.^1 3 10–17^ With these limitations, overall the available literature points towards a minimally increased risk in untreated patients with IBD^18^ and an increased risk among patients with IBD treated with immunomodulators, particularly thiopurine analogues.^18–21^ To what extent the disease activity itself or the use of different immunosuppressants drive an increased risk of lymphoma in IBD is still unclear.^21^

To add to the existing literature on risk of lymphoma in patients with IBD, we undertook a nationwide population study of IBD and lymphoma risk in Norway and Sweden. We took advantage of cross-linkage between numerous high-quality registries to empanel a large patient cohort with complete, long-term follow-up.

## Methods

### Study cohort

Norway and Sweden are countries with similar populations with regard to distribution of age, socioeconomic factors, ethnicity, and access to public healthcare systems. We defined our study population using data from databases of all public hospitals in Norway (from 1 January 1987 to 31 December 2015), and from the Swedish Patient Register (from 1 January 1993 to 31 December 2016).

We used the individually unique national registration numbers in registries in each country to link patients with IBD to the following public registries for data on outcomes and censoring: cancer (Cancer Registry of Norway, the Swedish Cancer Register), mortality (the Norwegian Cause of Death Registry, the Swedish Cause of Death Register), demographics and migration (the total Population Register in both countries). The empanelled registries are nationwide and entail compulsory, automated registration of all patients and are close to 100% complete.

Patients were followed from the date of diagnosis of IBD until diagnosis of lymphoma, diagnosis of any other cancer, emigration, death, or end of follow-up (Norway: 31 December 2015; Sweden: 31 December 2016), whichever occurred first. Patients with a cancer diagnosis prior to IBD diagnosis were excluded. A flow chart summarising the enrolment procedures is shown in online supplemental figure 1.

10.1136/bmjgast-2022-001037.supp1 Supplementary data

To ensure high specificity of IBD diagnosis, we required at least two inpatient and/or outpatient diagnosis codes of IBD. This approach yields a positive predictive value of an IBD diagnosis of 90% in our cohort.^22^ We used the International Classification of Disease (ICD) codes from the first two visits to define IBD subtypes at baseline: patients were classified as having ulcerative colitis (ICD-9: 556; ICD-10: K51) or Crohn’s disease (ICD-9: 555; ICD-10: K50) if their first two codes were for ulcerative colitis or Crohn’s disease, respectively, and unclassified IBD if their first two codes were indeterminate colitis (ICD-10 K52.3) or any combination of codes (online supplemental table 1). In main analyses, the date of the second diagnostic listing was defined as date of IBD onset.

### Comparator

The general population of Norway and Sweden served as reference, using anonymised individual data of all cancer diagnoses in Norway and Sweden diagnosed during the study period. The datasets include information on cancer topography and morphology, in addition to date of diagnosis, patient sex and birth year. Similar to the patients with IBD, we required that incident cases of lymphoma in the general population had no history of cancer.

### Covariates

In Norway, outpatient diagnoses became gradually available in hospital databases by geographic roll-out during the study period, while in Sweden, outpatient data from the whole country were available from 2001; patients diagnosed up until 2003 could represent a mix of patients with incident and prevalent IBD. We therefore analysed the data by calendar year periods of enrolment (1987/1993 to 2002 and 2003 to 2015/2016).

We used the Montreal classification to define extent of disease during follow-up, based on ICD-9 and ICD-10 codes.^23^ The most severe disease extension during follow-up was used to classify patients (online supplemental table 2). Indicators of disease severity such as laboratory markers, endoscopic or clinical scores were not available in our databases. We therefore used bowel surgery during follow-up as an indicator of disease severity (online supplemental table 3). Primary sclerosing cholangitis (PSC) was defined using ICD-9 (Norway: 5761; Sweden: 576B) and ICD-10 codes (Norway: K83.0; Sweden: K83.0). Bowel surgery and PSC were analysed as time-varying covariates.

### Pharmacotherapy

Data on pharmacotherapy were available for all Swedish patients from the Swedish Drug Register from 2005 onwards. We focused analyses on thiopurines and anti-tumour necrosis factor (TNF)-α therapy, as these are frequently used and have been associated with lymphoma risk in previous studies.^24^ The anatomical therapeutic chemical (ATC) codes are listed in online supplemental table 4. During each day of follow-up, patients were categorised as being exposed to one or two classes of drugs. Patients were considered unexposed until the day of their first prescription. For each drug, any overlapping prescriptions within a 90-day period were bridged, and duration of exposure estimated to last until end of the last prescription. Duration of prescriptions was assumed to last 3 months. To minimise prevalent user bias, we included a 1-year wash-out window and excluded patients with their first prescription in the year prior to cohort entry (their second IBD diagnosis). To eliminate any reverse causality due to initiation of treatment due to cancer symptoms, we introduced a 1-year lag between initiation of treatment and the follow-up window. Additionally, the discontinuation time between prescriptions (where the interval was longer than >3 months) was removed, since patients in the discontinuation period were different from patients without any treatment or with each class of the treatments. See online supplemental figure 2 for design diagram. Individual pharmacotherapy data were not available in Norway.

### Outcome

The cancer registries have different coding practices: in Norway, all cancer diagnoses are retrospectively coded according to ICD-O-3. In Sweden, ICD-O-2 and Systematized Nomenclature of Medicine (SNOMED) codes were introduced in the Swedish Cancer Register from 1 January 1993. To allow for accurate subgrouping of lymphomas, we restricted the IBD cohort in Sweden to patients diagnosed from that date (see online supplemental table 5 for outcome definitions in each country). Lymphomas were divided into NHL and HL, as the codes used in the Swedish Cancer Register do not allow adequate resolution to accurately define subcategories such as B-cell, T-cell or NK-cell lymphomas.

### Statistical analyses

We calculated crude incidence rates (IRs) for each type of lymphoma, and present IRs standardised for age and sex to the Norwegian population of 1987 graphically. Cumulative incidence was calculated by Kaplan-Meier method, using the cumulative hazard function as a direct estimate. Patients were considered at risk from 1 year after IBD onset to reduce bias due to reverse causality.

We calculated standardised incidence ratios (SIRs, the ratios of observed cancer cases divided by expected number of cancer cases in the population) with 95% CIs, overall and stratified by covariates, to estimate the relative risk of lymphomas in patients with IBD compared with the general population. The expected number was derived by multiplying the person-years at risk with country-specific, age-specific (5-year strata), sex-specific, and calendar year-specific IRs in the general population.

The Cochran-Armitage trend test was used to evaluate cancer risk across age groups and follow-up duration. SIRs using data from Norway and Sweden were analysed separately and combined.^25^

### Sensitivity analyses

In sensitivity analyses, patients were considered at risk from their second diagnostic listing of IBD (inpatient or outpatient), that is, the 1-year lag was removed.

### Patient and public involvement statement

Patients and/or the public were not involved in the design, or conduct, or reporting, or dissemination plans of this research.

## Results

### Patient characteristics

Our cohort consisted of 131 492 patients with IBD with a median follow-up of 9.6 years (IQR 4.3–14.9 years), and a total of 1 322 831 person-years of follow-up (table 1). In Sweden, 473 (1%) patients were included in analyses of anti-TNF-α therapy, 10 271 (25%) included in analyses of thiopurines and 3909 (10%) patients analysed as treated with a combination of these drugs since 2005 (after first year of follow-up).

**Table 1:** General characteristics of patients with IBD in Norway and Sweden, 1987–2016

Characteristics	Norway, 1987–2015	Sweden, 1993–2016	Total
Total, n	44 452	87 040	131 492
Total person-years	483 293	839 539	1 322 831
Median follow-up years (IQR)	9.9 (4.2–16.7)	9.3 (4.3–14.5)	9.6 (4.3–14.9)
Sex (n, %)			
Female	21 709 (48.8)	42 182 (48.5)	63 891 (48.5)
Male	22 743 (51.1)	44 858 (51.5)	67 601 (51.4)
Age (years) at IBD diagnosis (n, %)			
<20	5119 (11.5)	9516 (10.9)	14 635 (11.1)
20–39	17 523 (39.4)	30 680 (35.2)	48 203 (36.6)
40–59	13 514 (30.4)	28 212 (32.4)	41 726 (31.7)
60+	8296 (18.6)	18 632 (21.4)	26 928 (20.4)
Calendar year at IBD diagnosis* (n, %)			
1987/1993–2002	20 649 (46.4)	39 114 (44.9)	59 763 (45.4)
2003–2015/2016	23 803 (53.5)	47 926 (55.1)	71 729 (54.5)
IBD subtype (n %)			
Ulcerative colitis	27 214 (61.2)	51 130 (58.7)	78 344 (59.5)
Crohn’s disease	13 347 (30.0)	28 900 (33.2)	42 247 (32.1)
IBD unclassified	3891 (8.7)	7010 (8.1)	10 901 (8.2)
Extent of ulcerative colitis† (n, %)			
E1 (proctitis)	6007 (22.1)	6326 (12.4)	12 333 (15.7)
E2 (left-sided)	2380 (8.7)	10 836 (21.2)	13 216 (16.8)
E3 (extensive)	14 621 (53.7)	24 649 (48.2)	39 270 (50.1)
Ex (not defined)	4206 (15.4)	9319 (18.2)	13 525 (17.2)
Location of Crohn’s disease† (n, %)			
L1 (terminal ileitis)	3126 (23.4)	5768 (20.0)	8894 (21)
L2 (colonic)	2970 (22.2)	5666 (19.6)	8636 (20.4)
L3 (ileocecal)	5453 (40.8)	12 879 (44.6)	18 332 (43.3)
Lx (not defined)	1798 (13.4)	4587 (15.9)	6385 (15.1)
Behaviour of Crohn’s disease† (n, %)			
B1 (non-stricturing/non-penetrating)	10 392 (77.8)	21 789 (75.4)	32 181 (76.1)
B2 (stricturing)	1620 (12.1)	5138 (17.8)	6758 (15.9)
B3 (penetrating)	931 (6.9)	1092 (3.8)	2023 (4.7)
B2B3 (stricturing and penetrating)	404 (3.0)	881 (3.0)	1285 (3.0)
Perianal disease of Crohn’s disease† (n, %)			
	1991 (14.9)	5928 (20.5)	7919 (18.7)
Primary sclerosing cholangitis (n, %)			
	1035 (2.3)	3111 (3.6)	4146 (3.1)
Bowel surgery during follow-up‡ (n, %)			
	8828 (19.8)	10 392 (11.9)	19 220 (14.6)

*Patients diagnosed before 2002 could represent a mix of prevalent and incident patients with IBD as outpatient data were gradually included in the Norwegian hospital databases and the Swedish National Patient Register.

†Definitions and diagnostic codes were used according to the Montreal classifications using ICD-9 and ICD-10 in Norwegian data and ICD-10 in Swedish data representing maximum disease involvement during follow-up. See online supplemental table 2 for definitions.

‡Bowel surgeries included colectomy, small bowel resection, rectal resection, and colon resection during follow-up. See online supplemental table 3 for definitions.

IBD, inflammatory bowel disease; ICD, International Classification of Disease.

### Non-Hodgkin’s lymphoma

During follow-up, 369 patients were diagnosed with NHL, 231 patients with ulcerative colitis and 117 patients with Crohn’s disease (table 2).

**Table 2:** Crude incidence rates per 100 000 person-years, number of cancer cases and SIRs of non-Hodgkin's lymphoma and Hodgkin’s lymphoma in patients with IBD in Norway and Sweden, 1987–2016*

Parameters	Non-Hodgkin’s lymphoma			Hodgkin’s lymphoma		
	IR (95% CI)	O/E	SIR (95% CI)	IR (95% CI)	O/E	SIR (95% CI)
Total	30.8 (27.8 to 34.2)	369/277.8	1.3 (1.2 to 1.5)	3.7 (2.7 to 4.9)	44/28.5	1.5 (1.1 to 2.1)
Sex						
Female	24.8 (20.9 to 29.2)	145/109.4	1.3 (1.1 to 1.6)	2.7 (1.6 to 4.4)	16/11.6	1.4 (0.8 to 2.2)
Male	36.6 (32.0 to 41.7)	224/168.4	1.3 (1.2 to 1.5)	4.6 (3.0 to 6.6)	28/16.8	1.7 (1.1 to 2.4)
Age (years) at IBD diagnosis						
<20	5.9 (2.6 to 11.7)	8/2.1	3.9 (1.7 to 7.6)	3.0 (0.8 to 7.6)	4/4.3	0.9 (0.3 to 2.4)
20–39	9.8 (7.2 to 13.0)	48/28.7	1.7 (1.2 to 2.2)	3.5 (2.0 to 5.6)	17/11.7	1.5 (0.8 to 2.3)
40–59	39.5 (33.6 to 46.1)	161/122.8	1.3 (1.1 to 1.5)	3.2 (1.7 to 5.4)	13/8	1.6 (0.9 to 2.8)
60+	92.2 (78.1 to 08.1)	152/124.2	1.2 (1.0 to 1.4)	6.1 (2.9 to 11.2)	10/4.4	2.3 (1.1 to 4.2)
P for trend			0.119			0.330
Calendar year of IBD diagnosis†						
1987/1993–2002	32.1 (28.4 to 36.2)	268/198.4	1.4 (1.2 to 1.5)	3.5 (2.3 to 5.0)	29/19.1	1.5 (1.0 to 2.2)
2003–2015/2016	27.9 (22.7 to 33.9)	101/79.4	1.3 (1.0 to 1.5)	4.1 (2.3 to 6.8)	15/9.4	1.6 (0.9 to 2.6)
IBD subtype						
Ulcerative colitis	32.1 (28.1 to 36.5)	231/178.1	1.3 (1.1 to 1.5)	3.8 (2.5 to 5.5)	27/17.1	1.6 (1.0 to 2.3)
Crohn’s disease	29.4 (24.3 to 35.2)	117/83	1.4 (1.2 to 1.7)	4.0 (2.3 to 6.5)	16/9.5	1.7 (1.0 to 2.7)
IBD unclassified	26.8 (16.6 to 40.9)	21/16.7	1.3 (0.8 to 1.9)	1.3 (0.0 to 7.1)	1/1.9	0.5 (0.0 to 2.9)
Extent of ulcerative colitis						
E1 (proctitis)	30.7 (19.5 to 46.1)	23/17.9	1.3 (0.8 to 1.9)	2.7 (0.3 to 9.7)	2/1.7	1.2 (0.1 to 4.2)
E2 (left-sided)	31.2 (23.0 to 41.4)	48/39.5	1.2 (0.9 to 1.6)	5.9 (2.7 to 11.1)	9/3.5	2.5 (1.2 to 4.8)
E3 (extensive)	34.3 (27.6 to 42.1)	91/69.2	1.3 (1.1 to 1.6)	3.0 (1.3 to 5.9)	8/6.4	1.3 (0.5 to 2.5)
Ex (not defined)	30.6 (23.8 to 38.7)	69/51.5	1.3 (1.0 to 1.7)	3.5 (1.5 to 7.0)	8/5.4	1.5 (0.6 to 2.9)
Location of Crohn’s disease‡						
L1 (terminal ileitis)	34.4 (21.0 to 53.1)	20/15.5	1.3 (0.8 to 2.0)	1.7 (0.0 to 9.6)	1/1.4	0.7 (0.0 to 4.0)
L2 (colonic)	37.3 (24.6 to 54.2)	27/16.2	1.7 (1.1 to 2.4)	6.9 (2.2 to 16.1)	5/1.7	2.9 (0.9 to 6.8)
L3 (ileocecal)	27.5 (20.2 to 36.5)	47/35.9	1.3 (1.0 to 1.7)	3.5 (1.3 to 7.6)	6/4.0	1.5 (0.6 to 3.3)
Lx (not defined)	23.8 (15.1 to 35.8)	23/15.4	1.5 (0.9 to 2.2)	4.1 (1.1 to 10.6)	4/2.4	1.7 (0.5 to 4.3)
Behaviour of Crohn’s disease‡						
B1 (non-structuring/non-penetrating)	25.9 (20.2 to 32.6)	72/55.8	1.3 (1.0 to 1.6)	4.3 (2.2 to 7.5)	12/6.7	1.8 (0.9 to 3.1)
B2 (stricturing)	46.4 (32.7 to 63.9)	37/21.2	1.7 (1.2 to 2.4)	-	0/1.8	-
B3 (penetrating)	23.0 (7.5 to 53.7)	5/2.8	1.8 (0.6 to 4.1)	9.2 (1.1 to 33.3)	2/0.6	3.6 (0.4 to 13.0)
B2B3 (stricturing and penetrating)	16.5 (3.4 to 48.1)	3/3.2	0.9 (0.2 to 2.7)	11.0 (1.3 to 9.7)	2/0.4	4.7 (0.6 to 17.1)
Perianal disease of Crohn’s disease‡						
No	25.0 (19.7 to 31.2)	77/66.4	1.2 (0.9 to 1.4)	3.2 (1.6 to 6.0)	10/7.3	1.4 (0.7 to 2.5)
Yes	44.5 (31.8 to 60.5)	40/16.6	2.4 (1.7 to 3.3)	6.7 (2.4 to 14.5)	6/2.2	2.8 (1.0 to 6.1)
Years since IBD diagnosis						
>1–2	31.2 (21.9 to 43.0)	37/22.4	1.7 (1.2 to 2.3)	3.4 (0.9 to 8.6)	4/2.9	1.4 (0.4 to 3.6)
>2–5	28.1 (22.5 to 34.6)	88/63.3	1.4 (1.1 to 1.7)	1.6 (0.5 to 3.7)	5/7.5	0.7 (0.2 to 1.5)
>5–10	29.5 (24.3 to 35.4)	115/89.9	1.3 (1.1 to 1.5)	4.9 (2.9 to 7.6)	19/9.4	2.0 (1.2 to 3.2)
10+	34.5 (28.8 to 41.0)	129/102.3	1.3 (1.1 to 1.5)	4.3 (2.4 to 6.9)	16/8.7	1.8 (1.1 to 3.0)
P for trend			0.377			0.312
PSC§						
No	30.0 (26.9 to 33.3)	349/271.9	1.3 (1.2 to 1.4)	3.5 (2.5 to 4.8)	41/27.7	1.5 (1.1 to 2.0)
Yes	63.8 (39.0 to 98.6)	20/5.9	3.4 (2.1 to 5.2)	10.0 (2.1 to 9.3)	3/0.8	3.9 (0.8 to 11.4)
Bowel surgery during follow-up¶						
No	29.9 (26.6 to 33.5)	303/240.9	1.3 (1.1 to 1.4)	3.5 (2.4 to 4.8)	34/23.3	1.5 (1.0 to 2.0)
Yes	36.0 (27.8 to 45.8)	66/36.9	1.8 (1.4 to 2.3)	5.5 (2.6 to 10.0)	10/4.4	2.3 (1.1 to 4.1)

*All patients were at risk from 1 year after IBD diagnosis.

†Patients diagnosed before 2002 could represent a mix of prevalent and incident patients with IBD as outpatient data were gradually included in hospital databases and the Swedish national patient register.

‡Definitions and diagnostic codes were used according to the Montreal classifications using ICD-9 and ICD-10 in Norwegian data and using ICD-10 in Swedish data, representing maximum disease involvement during follow-up. See online supplemental table 2 for definitions.

§Patients with PSC contributed person-time to the non-PSC group until the date of PSC diagnosis.

¶Bowel surgeries included colectomy, small bowel resection, rectal resection and colon resection during follow-up. See online supplemental table 3 for definitions.

E, expected number of cases; IBD, inflammatory bowel disease; ICD, International Classification of Disease; IR, incidence ratio, per 100 000 person-years; O, observed number of cases; PSC, primary sclerosing cholangitis; SIR, standardised incidence ratio.

**Figure 1:**
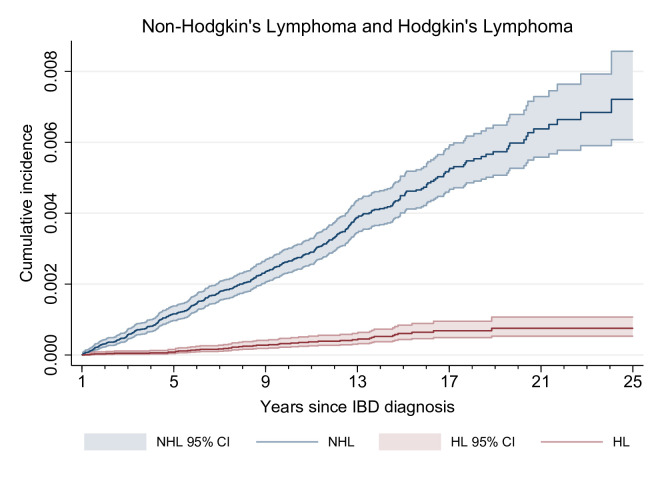
Cumulative incidence of non-Hodgkin’s lymphoma (NHL) and Hodgkin’s lymphoma (HL) by year since onset of inflammatory bowel disease (IBD). Patients were at risk after 1 year of follow-up. Cumulative incidence was calculated directly from Nelson-Aalen cumulative hazard estimates.

We observed a crude IR of NHL of 30.8 (95% CI 27.8 to 34.2) per 100 000 person-years, corresponding to three patients with lymphoma if 10 000 patients with IBD were followed for 1 year. We found an overall 30% excess risk as compared with the background population (SIR 1.3; 95% CI 1.2 to 1.5). Cumulative incidence of NHL was 0.26% after 10 years of follow-up (95% CI 0.23% to 0.30%) (figure 1).

**Figure 2:**
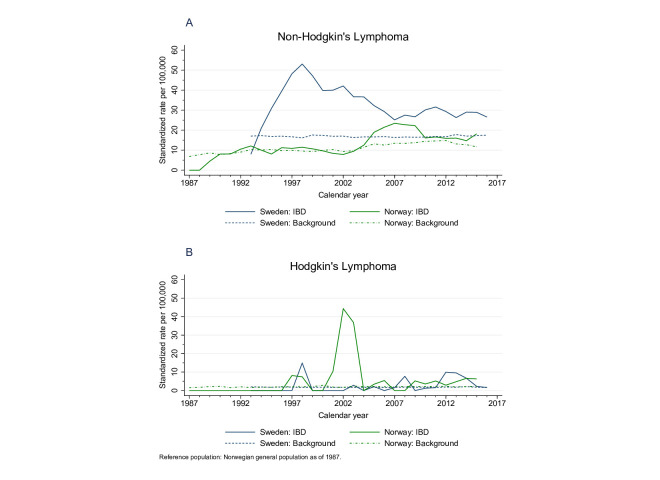
Age-standardised and sex-standardised incidence rates of non-Hodgkin's lymphoma (panel A) and Hodgkin’s lymphoma (panel B). The Norwegian background population of 1987 was used as reference. Panel A: the peak in 1997 in Sweden was due to cases of lymphoma in strata with few patients with inflammatory bowel disease (IBD). Moreover, the number of patients with incident IBD was artifactually low before 2001, when outpatient records became available in the Swedish Patient Register (online supplemental figure 2). Panel B: the figure is based on 14 cases in Norway and 29 cases in Sweden. The increased rates in 2002 and 2003 in Norway were due to two cases in strata with very few patients with IBD.

We found a higher relative risk, but low IRs with younger age of onset of IBD, while the IRs were highest in those diagnosed at a later age, with a lower relative risk compared to the background. While the combined results showed no difference between the two study periods (before and after 2002), in Norway, the excess risk was most pronounced in the second half of the study period, while in Sweden the results were opposite (online supplemental table 6). Compared with the general population of Norway, patients with IBD in Norway had similar age and sex SIRs of NHL until 2004, when an excess risk became evident. In Sweden, the excess risk was more pronounced in the earlier years of the study period and remained increased (figure 2, panel A).

Among patients with ulcerative colitis, the excess risk was about 30% regardless of extent of disease. Likewise, among patients with Crohn’s disease we found no apparent relation between location or extent of disease and the excess risk of NHL, except that those with perianal disease encountered a markedly increased risk (SIR 2.4; 95% CI 1.7 to 3.3). Of all categories in table 2, patients diagnosed with PSC experienced the highest excess risk (SIR 3.4; 95% CI 2.1 to 5.2). For patients who underwent bowel surgery, the risk was only slightly increased (SIR 1.8; 95% CI 1.4 to 2.3). Although we excluded the first year of follow-up to reduce selection bias, the overall excess risk was higher 1–2 years after diagnosis compared with subsequent years with follow-up beyond 10 years.

### Hodgkin’s lymphoma

We identified 44 incident cases of HL in patients with IBD; 27 in patients with ulcerative colitis and 16 in patients with Crohn’s disease. There was no clear pattern in IRs over time (figure 2, panel B). We found a crude IR of 3.7 (95% CI 2.7 to 4.9) per 100 000 person-years, corresponding to <1 case of lymphoma if 10 000 patients with IBD were followed for 1 year. Compared with the background, we found a 50% excess risk with an overall SIR of 1.5 (95% CI 1.1 to 2.1). The 10-year cumulative incidence of HL was 0.06% (95% CI 0.04% to 0.08%).

Rates and SIRs were higher among men and those diagnosed with IBD at or after 60 years of age. There were no significant differences for patients with perianal disease, PSC or bowel surgery. In Norway, the SIR was higher in the latter study period, while it was similar in the two periods in Sweden (online supplemental table 7).

### Pharmacotherapy

Among patients with IBD in Sweden, 14 653 (36%) were treated with thiopurines or anti-TNF-α therapy from 2005. The IR of NHL was 10.5 (95% CI 1.5 to 74.3) per 100 000 person-years for patients not treated with thiopurines or anti-TNF-α therapy (SIR 0.9; 95% CI 0.6 to 1.3) (table 3). For patients treated with thiopurines or anti-TNF-α therapy, SIRs for NHL were significantly increased among patients on combination therapy with both drug classes (SIR 5.7; 95% CI 2.7 to 11.9), while monotherapy with thiopurines also increased the risk of NHL with SIR 2.8 (95% CI 1.4 to 5.7). For HL, the overall IR was 5.0 (95% CI 2.0 to 10.4) per 100 000 person-years with an SIR of 1.9 (95% CI 0.9 to 4.0).

**Table 3:** Crude incidence rates per 100 000 person-years, number of cancer cases and SIRs of non-Hodgkin's lymphoma and Hodgkin’s lymphoma in patients with IBD in Sweden, 2005–2016*

Parameters	Non-Hodgkin’s lymphoma			Hodgkin’s lymphoma		
	IR (95% CI)	O/E	SIR (95% CI)	IR (95% CI)	O/E	SIR (95% CI)
Total	31.1 (23.2 to 41.8)	44/36.7	1.2 (0.9 to 1.6)	5.0 (2.0 to 10.4)	7/3.7	1.9 (0.9 to 4.0)
Immunosuppression therapy†						
No anti-TNF-α therapy or thiopurines	10.5 (1.5 to 74.3)	29/32.6	0.9 (0.6 to 1.3)	10.5 (1.5 to 74.4)	4/2.9	1.4 (0.5 to 3.6)
Monotherapy of anti-TNF-α	–	0/0.1	–	–	0/0	–
Monotherapy of thiopurines	46.5 (23.2 to 93.0)	8/2.8	2.8 (1.4 to 5.7)	11.6 (2.9 to 46.5)	2/0.5	4.2 (1.1 to 16.9)
Combined therapy	67.3 (32.1 to 141.2)	7/1.2	5.7 (2.7 to 11.9)	9.6 (1.4 to 68.4)	1/0.3	3.5 (0.5 to 24.8)

*The Swedish Prescribed Drug Register was available since 2005, therefore we included patients with IBD diagnosed from 2005; all patients were at risk from 1 year after initiation of treatment.

†Definitions of biological drugs and thiopurines according to Anatomical Therapeutic Chemical codes in the Swedish Prescribed Drug Register are shown in online supplemental table 4.

E, expected number of cases; IBD, inflammatory bowel disease; IR, incidence rate, per 100 000 person-years; O, observed number of cases; SIR, standardised incidence ratio; TNF, tumour necrosis factor.

### Sensitivity analysis

Analyses where the first year of follow-up was included showed similar results (online supplemental table 8 (NHL) and online supplemental table 9 (HL)).

## Discussion

Our nationwide study in Norway and Sweden showed a 30% increased risk of NHL in patients with ulcerative colitis and Crohn’s disease. This excess risk was of similar magnitude and in most instances statistically significant across numerous subgroups defined by patient and disease characteristics. The risk pattern was largely similar for HL, but was statistically insignificant, possibly due to smaller event rates and thus limited statistical power. The only compelling deviations from this pattern were more than threefold increased risk of lymphomas in patients with IBD with concomitant PSC and in those receiving combination immunosuppressive therapy with anti-TNF-α therapy and thiopurines.

Strengths of our study include its considerable size, prospective population-based design, complete long-term follow-up, and access to drug prescription data. We also tried to accommodate the substantial methodological challenges in studies of cancer risk in patients with IBD.^26^ Several limitations of our study are notable: both NHL and HL are complex, heterogenous disease entities with many subgroups; these subgroups likely have at least partly different causal patterns.^5 6^ Analyses of specific subgroups, particularly of NHL, may therefore be required for causal inference. Unfortunately, this would limit our follow-up time, since detailed classification is only available with the introduction of ICD-O-3 in the cancer registries, which was introduced in 2005 in Sweden. Furthermore, because the baseline incidence of HL is so low, even larger studies are needed to achieve informative statistical power. One important drawback of a registry-based approach is the lack of valid information on disease severity in register data. We used bowel surgery as a proxy for disease severity, but this may have low sensitivity and misclassify subgroups of patients with severe disease who did not undergo surgery. We also used the Montreal classification for disease severity, but the positive predictive value for extensive disease (pancolitis) seemed low, at only 60.8% in the Norwegian part of the cohort (data not shown). More importantly, disease severity could not be properly adjusted for in analyses on pharmacotherapy, which entails that we cannot exclude confounding by indication. Data on prescriptions were not available in the Norwegian part of the cohort, and as such the combined analyses with data from both countries represent a mix of treated and untreated patients with IBD. Finally, our analyses on anti-TNF-α therapy may be limited in power due to low sensitivity for certain agents in the Swedish Prescribed Drug Register and the Swedish National Patient Register, which could dilute any harmful effects due to misclassification of exposure.^27^

An excess of intestinal, mainly B-cell NHL, is widely accepted in Crohn’s disease,^28 29^ while only limited data exist on overall lymphoma risk in patients with IBD. Conclusions are often hampered by low statistical power, short follow-up, conflation of different haematopoietic malignancies and based on less than a dozen observed lymphomas among patients with IBD. Since the incidence of lymphoma is low, much larger studies are needed if the true excess risk is the order of 30% as suggested by our study. Comprising a large enough dataset with sufficient data on disease severity and pharmacotherapy poses a challenge. Based on our results, we believe that patients with IBD do experience a modestly increased risk of lymphomas compared with the background. High inflammatory activity has been suggested to be the main cause of increased lymphoma risk in autoimmune diseases.^4 30^ Our finding of higher excess risks in patients with concomitant PSC and perianal disease is in line with this theory. But we also acknowledge that causal inference requires more detailed and validated clinical data on disease severity, lymphoma subtype and location and lifestyle factors such as smoking and body mass index. With these limitations, we could not investigate the direct effect of immunosuppressive therapy on cancer risk.

The possible impact of immunosuppressive treatment on risk of lymphomas among patients with IBD has been extensively investigated, although published studies often suffer from low statistical power due to few cases.^8 21 31–33^ A larger cohort study based on 336 lymphoma cases comparing exposed and unexposed patients with IBD found a 2.6-fold increased lymphoma risk following thiopurine monotherapy, 2.4-fold increased risk following anti-TNF-α therapy and about 6-fold increased risk after combination therapy.^34^ We detected an increased risk associated with combination pharmacotherapy and use of thiopurines, in line with previous studies.^7 21 28^ We could not identify any cancers in patients exposed to anti-TNF-α monotherapy; the null finding is in line with another population-based study,^35^ which showed no association between anti-TNF-α therapy and ‘haematopoietic and lymphoid’ malignancies (adjusted rate ratio of 0.90 (95% CI 0.41 to 1.91)), although this finding was based on only eight cases.

In conclusion, we found consistent and statistically significant associations between IBD and malignant lymphomas with little evidence of heterogeneity across different patient and disease characteristics. The absolute risk is too low to warrant screening for lymphoma in patients with IBD and should prompt caretakers to emphasise the low absolute risk to patients in any shared decision-making process concerning choice of treatment for IBD. To strengthen the base for causal inference, future studies need to assess the associations separately for at least the major subtypes of both NHL and HL. They also need to investigate in detail the impact of immunosuppressive therapies, and the potential mutual confounding effect between disease severity and drug treatment; such studies require large numbers of observed cases and long-term follow-up, and more detailed data on disease severity than what is currently available in patient registers. A final challenge is to assess whether an increased risk of malignant lymphomas in IBD is indeed causal or arises due to shared risk factors, perhaps acting through immunological mechanisms or genetic predispositions.

## Data Availability

Data are not publicly available due to their containing information that could compromise the privacy of research participants. Summary data can be made available from the corresponding author on reasonable request.
