# Supplementary material for: Risk of malignant lymphomas in patients with inflammatory bowel disease: a population-based cohort study
Source: BMJ Open Gastroenterol. 2023 May 4;10(1):e001037. doi: 10.1136/bmjgast-2022-001037 (PMC10163486; doi:10.1136/bmjgast-2022-001037)
Supplement: Supplementary data [file bmjgast-2022-001037supp001.pdf]

Risk of malignant lymphomas in patients with inflammatory bowel disease: A population-based cohort study

Supplementary Appendix

Investigators:

Jingru Yu, Erle Refsum, Paulina Wieszczy, Lise M. Helsingen, Vera Perrin, Amanda Högdén, Magnus Løberg, Johannes Blom, Michael Bretthauer, Hans-Olov Adami, Weimin Ye, Mette Kalager.

Table of Contents

Fig. S1. Overview of cohort members included in analysis. 2

Fig. S2. Design diagram of analyses on pharmacotherapy in the Swedish cohort. 3

Table S1. ICD codes used to define patients with inflammatory bowel disease (IBD) from national patient registry and hospital databases. 4

Table S2. Definitions and diagnostic codes used to define ulcerative colitis (UC) and Crohn's disease (CD) according to the Montreal classification. 5

Table S3: Definition of IBD-related bowel surgeries in Norway and Sweden.[1] 7

Table S4: Definition of biologic drugs and thiopurines according to Anatomical Therapeutic Chemical (ATC) codes in the Swedish Prescribed Drug Register. 8

Table S5: Definition of cancer outcomes in Norway and Sweden.[2,3] 9

Table S6: Number of cases, standardized incidence ratio, and crude incidence rates per 100,000 person-years for non-Hodgkin lymphoma, Norway and Sweden. 10

Table S7: Number of cases, standardized incidence ratio, and crude incidence rates per 100,000 person-years for Hodgkin lymphoma, Norway and Sweden. 12

Table S8: Number of cases, standardized incidence ratio, and crude incidence rates per 100,000 person-years for non-Hodgkin lymphoma, Norway and Sweden, first year of follow-up included. 14

Table S9: Number of cases, standardized incidence ratio, and crude incidence rates per 100,000 person-years for Hodgkin lymphoma, Norway and Sweden, first year of follow-up included. 16

References 18

Fig. S1. Overview of cohort members included in analysis.

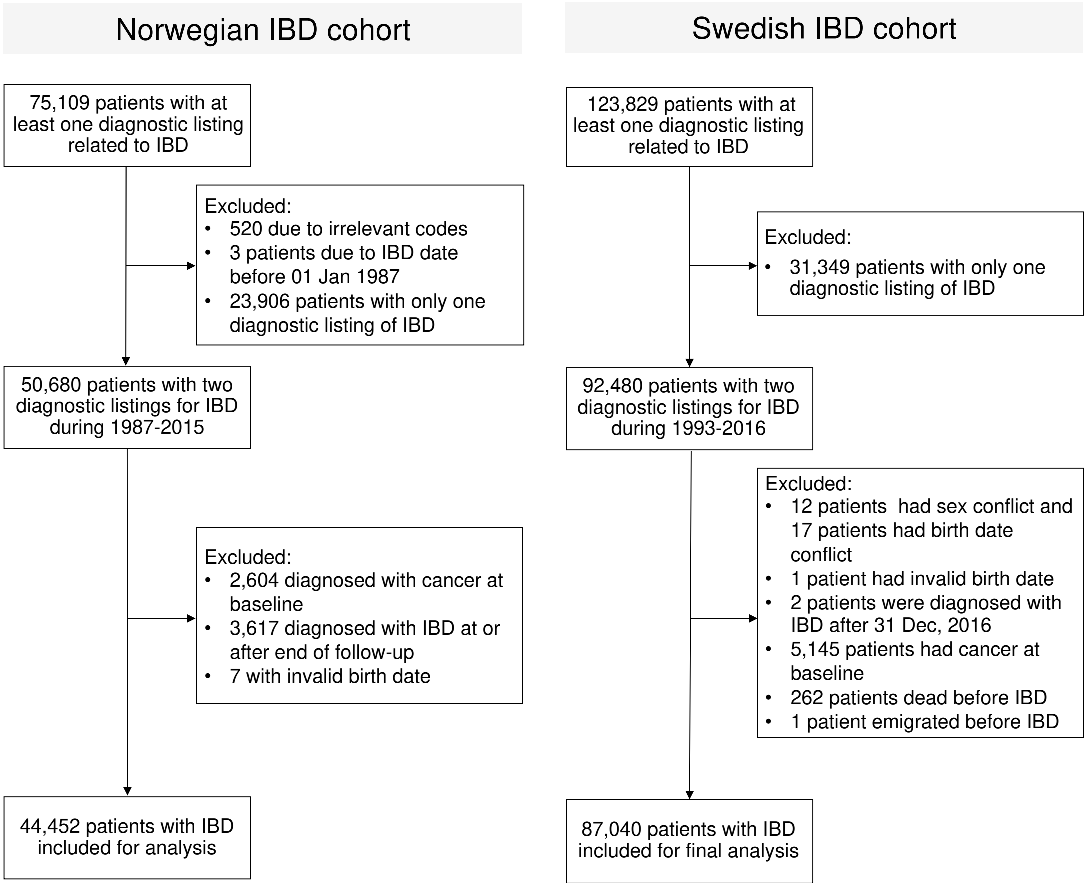

Fig. S2. Design diagram of analyses on pharmacotherapy in the Swedish cohort.
Diagram inspired by the work of Schneeweiss et al.,[1] made using template available at https://presc.sdu.dk/repeat-diagrams/

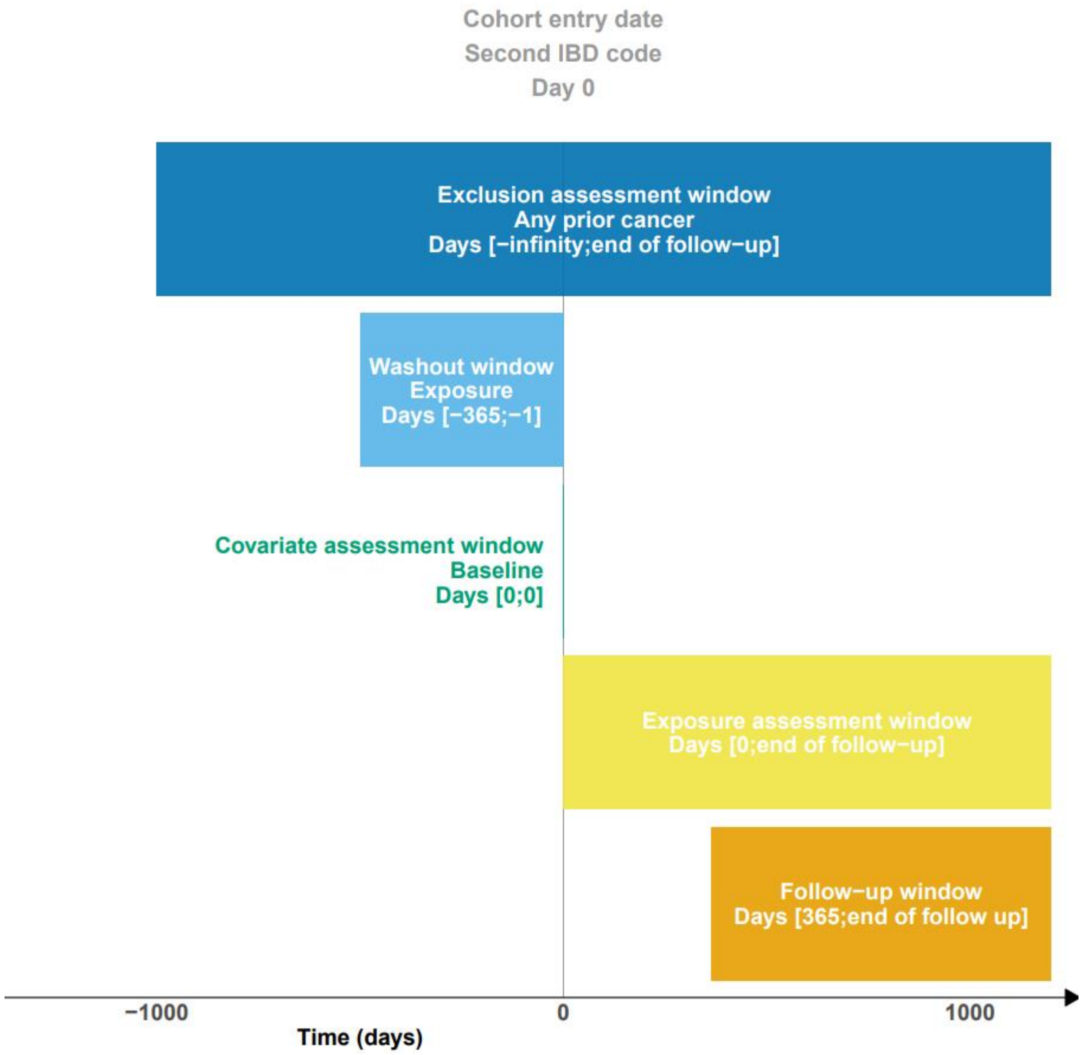

Yu J, *et al. BMJ Open Gastro* 2024; 10:e001037. doi: 10.1136/bmjgast-2022-001037

Table S1. ICD codes used to define patients with inflammatory bowel disease (IBD) from national patient registry and hospital databases.

**IBD subtype**	**ICD-9**	**ICD-10**
Ulcerative colitis (UC)	556	K51
Crohn's disease (CD)	555	K50
Unclassified (IBD-U)	UC+CD	UC+CD or K523

Classification of IBD subtypes is based on the two first diagnostic codes:
Ulcerative colitis, 1st code UC, 2nd code UC;
Crohn's disease, 1st code CD, 2nd code CD;
Unclassified, 1st code IBD-U, 2nd code IBD-U, or any combination of codes for UC, CD, and IBD-U.
ICD-9 was implemented in Sweden in 1987, and in Norway in 1986. ICD-10 was implemented in Sweden 1997, and in Norway in 1999.

Yu J, *et al. BMJ Open Gastro* 2024; 10:e001037. doi: 10.1136/bmjgast-2022-001037

Table S2. Definitions and diagnostic codes used to define ulcerative colitis (UC) and Crohn's disease (CD) according to the Montreal classification.

Ulcerative colitis	Extent	Diagnostic codes	
		**Norway**	**Sweden**
E1	Ulcerative proctitis	ICD-10=K512; ICD-9=5561	ICD-10=K512
E2	Left-sided UC	ICD-10=K513, K515; ICD-9=5565	ICD-10=K513, K515
E3	Extensive UC	ICD-10=K510, K511; ICD-9=5562	ICD-10=K510, K511
Ex	Extent not defined	ICD-10=K51, K514, K518, K519; ICD-9=5560, 55606, 5564, 5569	ICD-10=K514, K518, K519
**Crohn's disease**	**Location**	**Diagnostic codes**	
		**Norway**	**Sweden**
L1	Small bowel disease or terminal ileitis	ICD-10=K500; ICD-9=5550	ICD-10=K500; ICD-9=555A
L2	Colon	ICD-10=K501; ICD-9=5551, 55506	ICD-10=K501; ICD-9=555B
L3	Ileocecal Crohn's disease	ICD-10=K508; ICD-9=5552	ICD-10=K508; ICD-9=555C, 555AB
Lx	Location not defined	ICD-10=K509; ICD-9=555, 5559, 55502, 56301	ICD-10=K509; ICD-9=555X, 555
**Crohn's disease**	**Behaviour**	**Diagnostic codes**	
		**Norway**	**Sweden**
B1	Non-stricturing, non-penetrating	None of the ICD-codes for B2 or B3	None of the ICD-codes for B2 or B3
B2	Stricturing	Crohn's disease AND any of the following codes: ICD-10=K565, K566, K567, K624; ICD-9=5608, 5609	Crohn's disease AND any of the following codes: IC10=K565, K566, K567, K624; ICD-9=560W, 560X
B3	Penetrating	Crohn's disease AND any of the following diagnostic codes: ICD-10=K630, K632, K316, N823, N824; ICD-9=5374, 5696, 5961, 6191, 6298, OR any of the following surgical procedure codes (JHD20, JHD30, JHD33, JHD50, JHD60, JHD63, JFA76, JFA86, 4603, 4962)	Crohn's disease AND any of the following diagnostic codes: IC10=K630, K632, K316, N823, N824; ICD-9=569F, 537E, 596B, 619B, OR any of the following surgical procedure codes (JHD20, JHD30, JHD33, JHD50, JHD60, JHD63, JFA76, JFA86, no 4603 or2#GH 4962)
B2B3	Stricturing and penetrating	Both of the ICD codes for B2 and B3	Both of the ICD codes for B2 and B3
P	Perianal disease modifier	Crohn's disease AND any of the following diagnostic codes: K603, K604, K605, K610, K611, K612, K613, K614, K624; ICD-9=565, 5650, 5651, OR any of the following surgical procedure codes: (JHD20, JHD30, JHD33, JHD50, JHD60, JHD63, JHA00, JHA20, JHW96)	Crohn's disease AND any of the following diagnostic codes: K603, K604, K605, K610, K611, K612, K613, K614, K624; ICD-9=565B, 566, 569C, OR any of the following surgical procedure codes: (JHD20, JHD30, JHD33, JHD50, JHD60, JHD63, JHA00, JHA20, JHW96)

ICD-9 was implemented in Sweden in 1987, and in Norway in 1986. Swedish ICD-9 codes do not discriminate between different levels of extent of ulcerative colitis. ICD-10 was implemented in Sweden 1997, and in

Norway in 1999. All codes were captured in the Swedish National Patient Register and Norwegian hospital databases.

Table S3: Definition of IBD-related bowel surgeries in Norway and Sweden.[2]

	Norway		Sweden	
	**SIFF codes (1980-1998)**	**NCSP-N codes (1999- present)**	**5/6th revision (1963-1996)**	**7th revision (1997-present)**
Small bowel resection	4620, 4621, 4722, 4672	JFB00, JFB01, JFC00, JFC01, JFA60	4630, 4631	JFB00-01
Colon resection	464, 4723, 4673	JFB4, JFB50, JFB51, JFB6	464, 4713	JFB2-JFB6
Rectum resection	482	JGB	482	JGB
Other or combinations	4724	JFB20, JFB21, JFB33, JFB34, JFB53, JFB54, JFB96, JFB97		
Total colectomy	465	JFH	465	JFH

SIFF: Statens Institutt for Folkehelse; Norwegian Institute of Public Health classification of surgeries.
NCSP: Nordic Medico-Statistical Committee (NOMESCO) Classification of Surgical Procedures. NCSP was introduced in Norway in 1999, and in Sweden in 1997.

Table S4: Definition of biologic drugs and thiopurines according to Anatomical Therapeutic Chemical (ATC) codes in the Swedish Prescribed Drug Register.

Medication	ATC code
Thiopurines (mixed with Allopurinol or not)	
Azathioprine	L04AX01
Mercaptopurine	L01BB02
Anti-TNF agents	
Etanercept	L04AB01
Infliximab	L04AA12,
	L04AB02
Adalimumab	L04AB04
Certolizumab pegol	L04AB05
Golimumab	L04AB06

Yu J, *et al. BMJ Open Gastro* 2024; 10:e001037. doi: 10.1136/bmjgast-2022-001037

Table S5: Definition of cancer outcomes in Norway and Sweden.[3,4]

Table S5A: Outcome definitions in Norwegian data, using ICD-10 and ICD-O-3 codes.

Outcome	ICD-10	ICD-O-3 topography	ICD-O-3 morphology
Hodgkin's lymphoma	C81	C77	965-966
Non-Hodgkin's lymphoma			
B-cell	C82, C83, C85:	C77	967-969, 9728, 973, 9761-9767, 9769, 9823, 9826, 9833, 9836, 9940
CLL*	C91.1	C421	9823
T/NK-cell	C84, C86	C77	970, 971, 9729, 9827, 9831, 9834, 9837, 9948

*C91.1. CLL, leukemic presentation, morphology code 9823; C42.1. CLL, lymphoma presentation, morphology code 9823.

Table S5B. ICD-O-2 and SNOMED codes for leukemia and lymphomas in Sweden.

Outcome	ICD-O2	SNOMED
Non-Hodgkin's lymphoma (NHL)	C82-C85	Any code starting with 95, 96, 97,98
Hodgkin's lymphoma (HL)	C81	Any code starting with 95, 96, 97,98
Chronic lymphocytic leukemia	C91.1	Any code starting with C98

Table S6: Number of cases, standardized incidence ratio, and crude incidence rates per 100,000 person-years for non-Hodgkin lymphoma, Norway and Sweden.

Parameters	Norway 1987-2015			Sweden 1993-2016		
	IR (95% CI)	O/E	SIR (95% CI)	IR (95% CI)	O/E	SIR (95% CI)
Total	20,4 (16,6 to 25,1)	90/69,1	1,3 (1,1 to 1,6)	36,9 (32,8 to 41,5)	279/208,7	1,3 (1,2 to 1,5)
Sex						
Female	16,8 (12,1 to 23,3)	36/27	1,3 (1,0 to 1,8)	29,4 (24,4 to 35,5)	109/82,4	1,3 (1,1 to 1,6)
Male	23,8 (18,3 to 31,1)	54/42,1	1,3 (1,0 to 1,7)	44,1 (37,9 to 51,3)	170/126,3	1,3 (1,2 to 1,6)
Age at IBD diagnosis						
<20	1,9 (0,3 to 13,6)	1/0,6	1,8 (0,3 to 12,9)	8,5 (4,0 to 17,8)	7/1,5	4,6 (2,2 to 9,7)
20-39	6,5 (3,7 to 11,1)	13/10,3	1,3 (0,7 to 2,2)	12,2 (8,8 to 17)	35/18,4	1,9 (1,4 to 2,6)
40-59	32,8 (24,4 to 44,1)	44/32,6	1,3 (1,0 to 1,8)	42,7 (35,7 to 51,2)	117/90,2	1,3 (1,1 to 1,6)
60+	60,8 (43 to 85,9)	32/25,7	1,2 (0,9 to 1,8)	107 (89,5 to 127,9)	120/98,5	1,2 (1,0 to 1,5)
Calendar year of IBD diagnosis[a]						
1987/93-2002	19,3 (15,1 to 24,7)	63/51,4	1,2 (1,0 to 1,6)	40,4 (35,2 to 46,3)	205/147	1,4 (1,2 to 1,6)
2003-2015/6	23,7 (16,3 to 34,6)	27/17,8	1,5 (1,0 to 2,2)	29,8 (23,7 to 37,4)	74/61,7	1,2 (1,0 to 1,5)
IBD subtype						
Ulcerative colitis	14,8 (9,6 to 23,0)	20/15,7	1,3 (0,8 to 2,0)	36,9 (30,2 to 45)	97/67,3	1,4 (1,2 to 1,8)
Crohn's disease	23 (18 to 29,4)	63/48,6	1,3 (1,0 to 1,7)	37,7 (32,4 to 43,9)	168/129,5	1,3 (1,1 to 1,5)
IBD-unclassified	22,2 (10,6 to 46,5)	7/4,8	1,5 (0,7 to 3,1)	29,9 (17,7 to 50,4)	14/11,9	1,2 (0,7 to 2,0)
Extent of ulcerative colitis [b]						
E1 (proctitis)	25,6 (16,1 to 40,7)	18/13,3	1,3 (0,9 to 2,1)	38,5 (23,6 to 62,9)	16/10,8	1,5 (0,9 to 2,4)
E2 (left-sided)	26,8 (11,2 to 64,4)	5/3,7	1,3 (0,6 to 3,2)	35,9 (25,1 to 51,4)	30/26,2	1,1 (0,8 to 1,6)
E3 (extensive)	21,7 (15,4 to 30,6)	33/24,4	1,4 (1,0 to 1,9)	34,8 (28,2 to 43)	86/65,5	1,3 (1,1 to 1,6)
Ex (not defined)	21 (10,0 to 44,1)	7/7,1	1 (0,5 to 2,1)	49 (35,3 to 67,9)	36/27	1,3 (1,0 to 1,8)
Location of Crohn's disease[b]						
L1 (terminal ileitis)	33,9 (16,9 to 67,8)	8/3,1	2,6 (1,3 to 5,1)	44,3 (28,3 to 69,5)	19/13,2	1,4 (0,9 to 2,2)
L2 (colonic)	16,4 (6,8 to 39,3)	5/4,2	1,2 (0,5 to 2,8)	38,9 (24,8 to 61)	19/13,1	1,5 (0,9 to 2,3)
L3 (ileocecal)	9,1 (4,1 to 20,4)	6/6,1	1 (0,4 to 2,2)	29,9 (22,1 to 40,4)	42/31,7	1,3 (1,0 to 1,8)
Lx (not defined)	6,5 (0,9 to 46,5)	1/2,3	0,4 (0,1 to 3,1)	55,1 (34,2 to 88,6)	17/9,3	1,8 (1,1 to 2,9)
Behavior of Crohn's disease[b]						
B1 (non structuring / penetrating)	16,6 (10,2 to 27,1)	16/11,4	1,4 (0,9 to 2,3)	30,8 (23,7 to 40)	56/44,4	1,3 (1,0 to 1,6)
B2 (stricturing)	9,3 (2,3 to 37,3)	2/3	0,7 (0,2 to 2,7)	60 (43,1 to 83,6)	35/18,2	1,9 (1,4 to 2,7)
B3 (penetrating)	-	0/0,8	-	45,7 (19,0 to 109,9)	5/2,0	2,5 (1,0 to 5,9)
B2B3 (stricturing and penetrating)	31,5 (7,9 to 126,1)	2/0,6	3,5 (0,9 to 14,1)	8,4 (1,2 to 59,8)	1/2,6	0,4 (0,1 to 2,7)
Perianal disease of Crohn's disease[b]						
No	15,4 (9,6 to 24,8)	17/13,7	1,2 (0,8 to 2,0)	30,3 (23,6 to 39,1)	60/52,7	1,1 (0,9 to 1,5)
Yes	12,2 (3,9 to 37,8)	3/2	1,5 (0,5 to 4,7)	56,6 (41,0 to 78,1)	37/14,7	2,5 (1,8 to 3,5)
Years since IBD diagnosis						
>1 - 2	25,1 (13,5 to 46,6)	10/4,7	2,1 (1,1 to 3,9)	34,3 (23,5 to 49,9)	27/17,7	1,5 (1,0 to 2,2)
>2 - 5	21,9 (14,5 to 32,9)	23/13,4	1,7 (1,1 to 2,6)	31,3 (24,5 to 39,9)	65/49,9	1,3 (1,0 to 1,7)
>5 - 10	15 (9,7 to 23,2)	20/19,6	1 (0,7 to 1,6)	37 (30,2 to 45,2)	95/70,3	1,4 (1,1 to 1,7)
10+	22,8 (16,5 to 31,5)	37/31,4	1,2 (0,9 to 1,6)	43,4 (35,4 to 53,2)	92/70,9	1,3 (1,1 to 1,6)
Primary sclerosing cholangitis[c]						
No	19,7 (15,9 to 24,3)	85/68,1	1,2 (1,0 to 1,5)	36 (31,9 to 40,6)	264/203,8	1,3 (1,1 to 1,5)
Yes	59,5 (24,8 to 143,1)	5/1,1	4,7 (2,0 to 11,3)	65,4 (39,4 to 108,5)	15/4,8	3,1 (1,9 to 5,2)

Bowel surgery during follow-up[d]						
No	21,3 (17 to 26,7)	75/56,3	1,3 (1,1 to 1,7)	34,5 (30,3 to 39,3)	228/184,6	1,2 (1,1 to 1,4)
Yes	17,1 (10,3 to 28,3)	15/12,8	1,2 (0,7 to 1,9)	53,3 (40,5 to 70,2)	51/24,0	2,1 (1,6 to 2,8)

IR, incidence rate, per 100 000 person-years; CI, confidence interval; O, observed number of cases; E, expected number of cases; SIR, standardized incidence ratio.
[#]All patients were at risk from one year after IBD diagnosis;
[a]Patients diagnosed before 2002 could represent a mix of prevalent and incident patients with IBD, as outpatient data were gradually included in hospital databases and the Swedish national patient register;
[b]Definitions and diagnostic codes were used according to the Montreal classifications using ICD9 and ICD10 in Norwegian data and using ICD-10 in Swedish data, representing maximum disease involvement during follow-up, see Table S2 for details;
[c]Patients with primary sclerosing cholangitis contributed person-time to the non-PSC group until the date of PSC diagnosis;
[d]Bowel surgeries included colectomy, small bowel resection, rectal resection, and colon resection during follow-up. See Table S3 for definitions.

Yu J, *et al. BMJ Open Gastro* 2024; 10:e001037. doi: 10.1136/bmjgast-2022-001037

Table S7: Number of cases, standardized incidence ratio, and crude incidence rates per 100,000 person-years for Hodgkin lymphoma, Norway and Sweden.

Parameters	Norway 1987-2015			Sweden 1993-2016		
	IR (95% CI)	O/E	SIR (95% CI)	IR (95% CI)	O/E	SIR (95% CI)
Total	3,4 (2,1 to 5,6)	15/10,6	1,4 (0,9 to 2,3)	3,8 (2,7 to 5,5)	29/17,9	1,6 (1,1 to 2,3)
Sex						
Female	3,3 (1,6 to 6,9)	7/4	1,8 (0,8 to 3,7)	2,4 (1,3 to 4,7)	9/7,6	1,2 (0,6 to 2,3)
Male	3,5 (1,8 to 7,1)	8/6,6	1,2 (0,6 to 2,4)	5,2 (3,3 to 8,0)	20/10,2	2 (1,3 to 3,0)
Age at IBD diagnosis						
<20	3,8 (1 to 15,3)	2/1,7	1,2 (0,3 to 4,8)	2,4 (0,6 to 9,7)	2/2,7	0,7 (0,2 to 3)
20-39	3 (1,3 to 6,6)	6/5,1	1,2 (0,5 to 2,6)	3,8 (2,1 to 6,9)	11/6,6	1,7 (0,9 to 3)
40-59	3,7 (1,6 to 9)	5/2,6	1,9 (0,8 to 4,6)	2,9 (1,5 to 5,8)	8/5,4	1,5 (0,7 to 3)
60+	3,8 (0,9 to 15,2)	2/1,2	1,6 (0,4 to 6,6)	7,1 (3,6 to 14,3)	8/3,2	2,5 (1,3 to 5)
Calendar year of IBD diagnosis[a]						
1987/93-2002	2,4 (1,2 to 4,9)	8/7,7	1,0 (0,5 to 2,1)	4,1 (2,7 to 6,3)	21/11,4	1,8 (1,2 to 2,8)
2003-2015/6	6,1 (2,9 to 12,9)	7/2,9	2,4 (1,1 to 5,1)	3,2 (1,6 to 6,4)	8/6,5	1,2 (0,6 to 2,5)
IBD subtype						
Ulcerative colitis	3,7 (1,5 to 8,9)	5/3,4	1,5 (0,6 to 3,6)	4,2 (2,3 to 7,5)	11/6,1	1,8 (1,0 to 3,2)
Crohn's disease	3,6 (2,0 to 6,8)	10/6,5	1,5 (0,8 to 2,9)	3,8 (2,4 to 6,1)	17/10,6	1,6 (1,0 to 2,6)
IBD-unclassified	-	0/0,8	-	2,1 (0,3 to 15,2)	1/1,2	0,9 (0,1 to 6,2)
Extent of ulcerative colitis [b]						
E1 (proctitis)	7,1 (3,0 to 17,1)	5/1,6	3,1 (1,3 to 7,5)	4,8 (1,2 to 19,3)	2/0,9	2,1 (0,5 to 8,4)
E2 (left-sided)	-	0/0,4	-	4,8 (1,8 to 12,8)	4/1,9	2,1 (0,8 to 5,5)
E3 (extensive)	3,3 (1,4 to 7,9)	5/3,7	1,4 (0,6 to 3,2)	3,2 (1,6 to 6,5)	8/5,9	1,3 (0,7 to 2,7)
Ex (not defined)	-	0/0,8	-	4,1 (1,3 to 12,7)	3/1,7	1,7 (0,6 to 5,4)
Location of Crohn's disease[b]						
L1 (terminal ileitis)	-	0/0,6	-	2,3 (0,3 to 16,6)	1/1,0	1 (0,1 to 7,0)
L2 (colonic)	3,3 (0,5 to 23,2)	1/0,7	1,4 (0,2 to 9,7)	10,2 (4,3 to 24,6)	5/1,1	4,4 (1,8 to 10,5)
L3 (ileocecal)	6,1 (2,3 to 16,3)	4/1,7	2,4 (0,9 to 6,3)	3,6 (1,5 to 8,5)	5/3,3	1,5 (0,6 to 3,7)
Lx (not defined)	-	0/0,4	-	-	0/0,7	-
Behavior of Crohn's disease[b]						
B1 (non structuring / penetrating)	4,1 (1,6 to 11,1)	4/2,4	1,7 (0,6 to 4,5)	4,4 (2,2 to 8,8)	8/4,3	1,9 (0,9 to 3,7)
B2 (stricturing)	-	0/0,5	-	-	0/1,3	-
B3 (penetrating)	9,3 (1,3 to 65,9)	1/0,3	3,5 (0,5 to 24,6)	9,1 (1,3 to 64,9)	1/0,3	3,7 (0,5 to 26,4)
B2B3 (stricturing and penetrating)	-	0/0,2	-	16,8 (4,2 to 67,3)	2/0,3	7,8 (1,9 to 31,1)
Perianal disease of Crohn's disease[b]						
No	4,5 (1,9 to 10,9)	5/2,7	1,8 (0,8 to 4,4)	2,5 (1,1 to 6,1)	5/4,6	1,1 (0,5 to 2,6)
Yes	-	0/0,6	-	9,2 (4,1 to 20,4)	6/1,5	4 (1,8 to 8,9)
Years since IBD diagnosis						
>1 - 2	7,5 (2,4 to 23,3)	3/1	3 (1,0 to 9,4)	1,3 (0,2 to 9)	1/1,9	0,5 (0,1 to 3,8)
>2 - 5	1,9 (0,5 to 7,6)	2/2,6	0,8 (0,2 to 3,1)	1,4 (0,5 to 4,5)	3/4,9	0,6 (0,2 to 1,9)
>5 - 10	4,5 (2,0 to 10,0)	6/3,3	1,8 (0,8 to 4,1)	5,1 (2,9 to 8,7)	13/6,1	2,1 (1,2 to 3,7)
10+	2,5 (0,9 to 6,6)	4/3,7	1,1 (0,4 to 2,9)	5,7 (3,2 to 10)	12/5,0	2,4 (1,4 to 4,2)
Primary sclerosing cholangitis[c]						
No	3 (1,7 to 5,2)	13/10,4	1,3 (0,7 to 2,2)	3,8 (2,6 to 5,5)	28/17,3	1,6 (1,1 to 2,3)
Yes	23,8 (6,0 to 95,2)	2/0,2	9,1 (2,3 to 36,3)	4,6 (0,7 to 32,9)	1/0,5	1,8 (0,3 to 13,0)

Bowel surgery during follow-up[d]							
	No	3,7 (2,1 to 6,3)	13/8,5	1,5 (0,9 to 2,6)	3,3 (2,2 to 5,1)	21/14,9	1,4 (0,9 to 2,2)
	Yes	2,3 (0,6 to 9,1)	2/2,2	0,9 (0,2 to 3,7)	8,4 (4,2 to 16,7)	8/2,3	3,5 (1,8 to 7,0)

IR, incidence rate, per 100 000 person-years; CI, confidence interval; O, observed number of cases; E, expected number of cases; SIR, standardized incidence ratio.

[#]All patients were at risk from one year after IBD diagnosis;

[a]Patients diagnosed before 2002 could represent a mix of prevalent and incident patients with IBD, as outpatient data were gradually included in hospital databases and the Swedish national patient register;

[b]Definitions and diagnostic codes were used according to the Montreal classifications using ICD9 and ICD10 in Norwegian data and using ICD-10 in Swedish data, representing maximum disease involvement during follow-up, see Table S2 for details;

[c]Patients with primary sclerosing cholangitis contributed person-time to the non-PSC group until the date of PSC diagnosis;

[d]Bowel surgeries included colectomy, small bowel resection, rectal resection, and colon resection during follow-up. See Table S3 for definitions.

Table S8: Number of cases, standardized incidence ratio, and crude incidence rates per 100,000 person-years for non-Hodgkin lymphoma, Norway and Sweden, first year of follow-up included.

Parameters	Norway 1987-2015			Sweden 1993-2016		
	IR (95% CI)	O/E	SIR (95% CI)	IR (95% CI)	O/E	SIR (95% CI)
Total	20,1 (16,4 to 24,5)	97/74	1,3 (1,1 to 1,6)	41,2 (37,1 to 45,8)	346/227,0	1,5 (1,4 to 1,7)
Sex						
Female	17 (12,5 to 23,2)	40/29	1,4 (1 to 1,9)	31,2 (26,2 to 37,0)	128/89,6	1,4 (1,2 to 1,7)
Male	23 (17,7 to 29,8)	57/45	1,3 (1 to 1,6)	50,9 (44,5 to 58,1)	218/137,3	1,6 (1,4 to 1,8)
Age at IBD diagnosis						
<20	3,5 (0,9 to 14)	2/0,6	3,5 (0,9 to 13,9)	10,9 (5,9 to 20,2)	10/1,6	6,1 (3,3 to 11,4)
20 to 39	6 (3,5 to 10,2)	13/10,5	1,2 (0,7 to 2,1)	12,9 (9,5 to 17,6)	41/19,3	2,1 (1,6 to 2,9)
40-59	31,2 (23,4 to 41,7)	46/34,1	1,3 (1 to 1,8)	46,5 (39,4 to 54,8)	140/95,0	1,5 (1,2 to 1,7)
60+	59,7 (43 to 82,7)	36/28,8	1,2 (0,9 to 1,7)	119,7 (102,3 to 140,1)	155/111,1	1,4 (1,2 to 1,6)
Calendar year of IBD diagnosis[a]						
1987/93-2002	19 (14,9 to 24,2)	66/53,3	1,2 (1 to 1,6)	44,0 (38,7 to 49,9)	240/155,5	1,5 (1,4 to 1,8)
2003-2015/6	22,8 (16 to 32,4)	31/20,8	1,5 (1,1 to 2,1)	36,1 (29,8 to 43,7)	106/71,5	1,5 (1,2 to 1,8)
IBD subtype						
Ulcerative colitis	15,6 (10,3 to 23,4)	23/16,8	1,4 (0,9 to 2,1)	44,3 (37,3 to 52,7)	129/72,9	1,8 (1,5 to 2,1)
Crohn's disease	22,3 (17,6 to 28,4)	67/52	1,3 (1 to 1,6)	40,4 (35,2 to 46,4)	200/140,7	1,4 (1,2 to 1,6)
IBD to unclassified	19,9 (9,5 to 41,6)	7/5,2	1,3 (0,6 to 2,8)	31,8 (19,8 to 51,1)	17/13,4	1,3 (0,8 to 2,0)
Extent of ulcerative colitis [b]						
E1 (proctitis)	25 (15,9 to 39,2)	19/14,1	1,3 (0,9 to 2,1)	33,6 (20,6 to 54,9)	16/12,0	1,3 (0,8 to 2,2)
E2 (left-sided)	28,7 (12,9 to 63,9)	6/4,1	1,5 (0,7 to 3,3)	35,1 (25,0 to 49,4)	33/28,8	1,1 (0,8 to 1,6)
E3 (extensive)	20,5 (14,6 to 28,6)	34/26	1,3 (0,9 to 1,8)	36,9 (30,3 to 44,9)	100/70,0	1,4 (1,2 to 1,7)
Ex (not defined)	21,5 (10,8 to 43)	8/7,8	1 (0,5 to 2,1)	62,0 (47,1 to 81,6)	51/30,0	1,7 (1,3 to 2,2)
Location of Crohn's disease[b]						
L1 (terminal ileitis)	33,9 (17,6 to 65,2)	9/3,4	2,6 (1,4 to 5)	62,0 (43,4 to 88,7)	30/14,6	2,1 (1,4 to 2,9)
L2 (colonic)	18 (8,1 to 40)	6/4,5	1,3 (0,6 to 2,9)	42,3 (28,1 to 63,7)	23/14,2	1,6 (1,1 to 2,4)
L3 (ileocecal)	9,9 (4,7 to 20,7)	7/6,3	1,1 (0,5 to 2,3)	33,9 (25,9 to 44,5)	52/33,6	1,5 (1,2 to 2,0)
Lx (not defined)	5,9 (0,8 to 41,9)	1/2,5	0,4 (0,1 to 2,8)	68,3 (45,8 to 101,9)	24/10,5	2,3 (1,5 to 3,4)
Behavior of Crohn's disease[b]						
B1 (non structuring / penetrating)	17,9 (11,4 to 28)	19/12,3	1,5 (1 to 2,4)	40,9 (33,0 to 50,7)	83/48,4	1,7 (1,4 to 2,1)
B2 (stricturing)	8,7 (2,2 to 34,7)	2/3,1	0,6 (0,2 to 2,6)	63,2 (46,3 to 86,1)	40/19,5	2,1 (1,5 to 2,8)
B3 (penetrating)	0	0/0,8	- to 0)	41,6 (17,3 to 100,1)	5/2,2	2,3 (1,0 to 5,5)
B2B3 (stricturing and penetrating)	29,7 (7,4 to 118,6)	2/0,6	3,4 (0,9 to 13,6)	7,8 (1,1 to 55,7)	1/2,8	0,4 (0,1 to 2,6)
Perianal disease of Crohn's disease[b]						
No	16,5 (10,6 to 25,6)	20/14,7	1,4 (0,9 to 2,1)	40,0 (32,5 to 49,3)	88/57,3	1,5 (1,2 to 1,9)
Yes	11,3 (3,6 to 35)	3/2,1	1,5 (0,5 to 4,5)	57,6 (42,4 to 78,2)	41/15,6	2,6 (1,9 to 3,6)
Years since IBD diagnosis						
0 - 1	16,4 (7,8 to 34,4)	7/4,9	1,4 (0,7 to 3)	79,9 (62,9 to 101,5)	67/18,3	3,7 (2,9 to 4,7)

>1 - 2	25,1 (13,5 to 46,6)	10/4,7	2,1 (1,1 to 3,9)	34,3 (23,5 to 49,9)	27/17,7	1,5 (1,0 to 2,2)
>2 - 5	21,9 (14,5 to 32,9)	23/13,4	1,7 (1,1 to 2,6)	31,3 (24,5 to 39,9)	65/49,9	1,3 (1,0 to 1,7)
>5 - 10	15 (9,7 to 23,2)	20/19,6	1 (0,7 to 1,6)	37,0 (30,2 to 45,2)	95/70,3	1,4 (1,1 to 1,7)
10+	22,8 (16,5 to 31,5)	37/31,4	1,2 (0,9 to 1,6)	43,4 (35,4 to 53,2)	92/70,9	1,3 (1,1 to 1,6)
Primary sclerosing cholangitis[c]						
No	19,4 (15,8 to 23,8)	92/72,9	1,3 (1 to 1,5)	40,0 (35,9 to 44,6)	326/221,9	1,5 (1,3 to 1,6)
Yes	56,6 (23,6 to 136)	5/1,1	4,6 (1,9 to 10,9)	81,8 (52,8 to 126,8)	20/5,1	3,9 (2,5 to 6,1)
Bowel surgery during follow to up[d]						
No	20,4 (16,4 to 25,4)	80/60,8	1,3 (1,1 to 1,6)	38,7 (34,5 to 43,5)	287/202,4	1,4 (1,3 to 1,6)
Yes	18,7 (11,6 to 30,1)	17/13,2	1,3 (0,8 to 2,1)	60,0 (46,5 to 77,5)	59/24,6	2,4 (1,9 to 3,1)

IR, incidence rate, per 100 000 person-years; CI, confidence interval; O, observed number of cases; E, expected number of cases; SIR, standardized incidence ratio,

[#]All patients were at risk from one year after IBD diagnosis;

[a]Patients diagnosed before 2002 could represent a mix of prevalent and incident patients with IBD, as outpatient data were gradually included in hospital databases and the Swedish national patient register;

[b]Definitions and diagnostic codes were used according to the Montreal classifications using ICD9 and ICD10 in Norwegian data and using ICD-10 in Swedish data, representing maximum disease involvement during follow-up, see Table S2 for details;

[c]Patients with primary sclerosing cholangitis contributed person-time to the non-PSC group until the date of PSC diagnosis;

[d]Bowel surgeries included colectomy, small bowel resection, rectal resection, and colon resection during follow-up, See Table S3 for definitions,

Table S9: Number of cases, standardized incidence ratio, and crude incidence rates per 100,000 person-years for Hodgkin lymphoma, Norway and Sweden, first year of follow-up included.

**Parameters**	**Norway 1987-2015**			**Sweden 1993-2016**		
	**IR (95% CI)**	**O/E**	**SIR (95% CI)**	**IR (95% CI)**	**O/E**	**SIR (95% CI)**
Total	3,5 (2,2 to 5,7)	17/11,7	1,5 (0,9 to 2,3)	4,0 (2,9 to 5,7)	34/19,8	1,7 (1,2 to 2,4)
Sex						
Female	3 (1,4 to 6,2)	7/4,4	1,6 (0,8 to 3,3)	2,7 (1,5 to 4,8)	11/8,5	1,3 (0,7 to 2,3)
Male	4 (2,2 to 7,5)	10/7,3	1,4 (0,7 to 2,6)	5,4 (3,6 to 8,1)	23/11,3	2,0 (1,3 to 3,1)
Age at IBD diagnosis						
<20	3,5 (0,9 to 14)	2/1,8	1,1 (0,3 to 4,5)	4,4 (1,6-11,6)	4/2,9	1,4 (0,5 to 3,7)
20 to 39	3,2 (1,5 to 6,7)	7/5,7	1,2 (0,6 to 2,6)	3,8 (2,2 to 6,7)	12/7,5	1,6 (0,9 to 2,8)
40-59	4,1 (1,8 to 9,1)	6/2,9	2,1 (0,9 to 4,7)	2,7 (1,3 to 5,3)	8/5,8	1,4 (0,7 to 2,7)
60+	3,3 (0,8 to 13,3)	2/1,4	1,4 (0,4 to 5,7)	7,7 (4,2 to 14,4)	10/3,6	2,8 (1,5 to 5,1)
Calendar year of IBD diagnosis[a]						
1987/93-2002	2,6 (1,3 to 5)	9/8,2	1,1 (0,6 to 2,1)	4,2 (2,8 to 6,3)	23/12,2	1,9 (1,2 to 2,8)
2003-2015/6	5,9 (2,9 to 11,7)	8/3,5	2,3 (1,2 to 4,6)	3,7 (2,1 to 6,8)	11/7,6	1,4 (0,8 to 2,6)
IBD subtype						
Ulcerative colitis	3,4 (1,4 to 8,1)	5/3,7	1,4 (0,6 to 3,3)	4,8 (2,8 to 8,1)	14/6,8	2,1 (1,2 to 3,5)
Crohn's disease	3,7 (2 to 6,6)	11/7,1	1,5 (0,9 to 2,8)	3,8 (2,4 to 6,0)	19/11,7	1,6 (1,0 to 2,5)
IBD-unclassified	2,8 (0,4 to 20,1)	1/0,9	1,2 (0,2 to 8,3)	1,9 (0,3 to 13,3)	1/1,3	0,8 (0,1 to 5,4)
Extent of ulcerative colitis [b]						
E1 (proctitis)	6,6 (2,7 to 15,8)	5/1,7	2,9 (1,2 to 6,9)	4,2 (1,1 to 16,8)	2/1,1	1,8 (0,5 to 7,3)
E2 (left-sided)	-	0/0,5	-	5,3 (2,2 to 12,8)	5/2,2	2,3 (0,9 to 5,5)
E3 (extensive)	3,6 (1,6 to 8)	6/4,1	1,5 (0,7 to 3,3)	3,0 (1,5 to 5,9)	8/6,5	1,2 (0,6 to 2,5)
Ex (not defined)	-	0/0,8	-	4,9 (1,8 to 13,0)	4/1,9	2,1 (0,8 to 5,5)
Location of Crohn's disease[b]						
L1 (terminal ileitis)	-	0/0,7	-	4,1 (1,0 to 16,5)	2/1,1	1,7 (0,4 to 7,0)
L2 (colonic)	3 (0,4 to 21,3)	1/0,8	1,2 (0,2 to 8,8)	11,0 (5,0 to 24,6)	6/1,3	4,7 (2,1 to 10,5)
L3 (ileocecal)	5,6 (2,1 to 15)	4/1,8	2,2 (0,8 to 5,9)	3,9 (1,8 to 8,7)	6/3,6	1,7 (0,8 to 3,8)
Lx (not defined)	-	0/0,4	-	-	0/0,8	-
Behavior of Crohn's disease[b]						
B1 (non structuring / penetrating)	3,8 (1,4 to 10)	4/2,6	1,5 (0,6 to 4)	5,4 (3,0-9,8)	11/4,8	2,3 (1,3-4,1)
B2 (stricturing)	-	0/0,6	-	-	0/1,4	-
B3 (penetrating)	8,6 (1,2 to 60,7)	1/0,3	3,2 (0,4 to 22,6)	8,3 (1,2 to 59,1)	1/0,3	3,4 (0,5 to 24,0)
B2B3 (stricturing and penetrating)	-	0/0,2	-	15,7 (3,9 to 62,8)	2/0,3	7,2 (1,8 to 29,0)
Perianal disease of Crohn's disease[b]						
No	4,1 (1,7 to 9,9)	5/3	1,7 (0,7 to 4)	3,6 (1,8 to 7,3)	8/5,1	1,6 (0,8 to 3,1)
Yes	-	0/0,7	-	8,4 (3,8 to 18,8)	6/1,6	3,6 (1,6 to 8,1)
Years since IBD diagnosis						
0-1	4,7 (1,2 to 18,8)	2/1,1	1,9 (0,5 to 7,5)	6,0 (2,5 to 14,3)	5/2,0	2,5 (1,0 to 6,0)
>1 - 2	7,5 (2,4 to 23,3)	3/1	3 (1 to 9,4)	1,3 (0,2 to 9,0)	1/1,9	0,5 (0,1 to 3,8)
>2 - 5	1,9 (0,5 to 7,6)	2/2,6	0,8 (0,2 to 3,1)	1,4 (0,5 to 4,5)	3/4,9	0,6 (0,2 to 1,9)
>5 - 10	4,5 (2 to 10)	6/3,3	1,8 (0,8 to 4,1)	5,1 (2,9 to 8,7)	13/6,1	2,1 (1,2 to 3,7)
10+	2,5 (0,9 to 6,6)	4/3,7	1,1 (0,4 to 2,9)	5,7 (3,2 to 10,0)	12/5,0	2,4 (1,4 to 4,2)
Primary sclerosing cholangitis[c]						
No	3,2 (1,9 to 5,2)	15/11,4	1,3 (0,8 to 2,2)	4,0 (2,9 to 5,7)	33/19,2	1,7 (1,2 to 2,4)

Yes	22,6 (5,7 to 90,5)	2/0,2	8,7 (2,2 to 34,6)	4,1 (0,6 to 29,0)	1/0,6	1,6 (0,2 to 11,5)
Bowel surgery during follow to up[d]						
No	3,6 (2,1 to 6)	14/9,4	1,5 (0,9 to 2,5)	3,5 (2,4 to 5,2)	26/17,5	1,5 (1,0 to 2,2)
Yes	3,3 (1,1 to 10,2)	3/2,2	1,3 (0,4 to 4,2)	8,1 (4,1 to 16,3)	8/2,3	3,4 (1,7 to 6,8)

IR, incidence rate, per 100 000 person-years; CI, confidence interval; O, observed number of cases; E, expected number of cases; SIR, standardized incidence ratio,
[#]All patients were at risk from one year after IBD diagnosis;
[a]Patients diagnosed before 2002 could represent a mix of prevalent and incident patients with IBD, as outpatient data were gradually included in hospital databases and the Swedish national patient register;
[b]Definitions and diagnostic codes were used according to the Montreal classifications using ICD9 and ICD10 in Norwegian data and using ICD-10 in Swedish data, representing maximum disease involvement during follow-up, see Table S2 for details;
[c]Patients with primary sclerosing cholangitis contributed person-time to the non-PSC group until the date of PSC diagnosis;
[d]Bowel surgeries included colectomy, small bowel resection, rectal resection, and colon resection during follow-up, See Table S3 for definitions,

Yu J, *et al. BMJ Open Gastro* 2024; 10:e001037. doi: 10.1136/bmjgast-2022-001037

References

1. Schneeweiss S, Rassen JA, Brown JS, et al. Graphical Depiction of Longitudinal Study Designs in Health Care Databases. Ann Intern Med 2019;170(6):398-406. DOI: 10.7326/M18-3079.
2. Olen O, Askling J, Sachs MC, et al. Mortality in adult-onset and elderly-onset IBD: a nationwide register-based cohort study 1964-2014. Gut 2020;69(3):453-461. DOI: 10.1136/gutjnl-2018-317572.
3. Swerdlow SH, Campo E, Pileri SA, et al. The 2016 revision of the World Health Organization classification of lymphoid neoplasms. Blood 2016;127(20):2375-90. DOI: 10.1182/blood-2016-01-643569.
4. Fritz AG. International classification of diseases for oncology : ICD-O. Third edition, First revision. ed. Geneva: World Health Organization, 2013.
